# Rhizotomy targeting the intermediate nerve, the glossopharyngeal nerve and the upper 1st to 2nd rootlets of the vagus nerve for the treatment of laryngeal neuralgia combined with intermediate nerve neuralgia-a case report

**DOI:** 10.1186/1471-2482-14-60

**Published:** 2014-08-28

**Authors:** Qiang Zong, Kai Zhang, Guangliang Han, Shengye Yang, Lijiang Wang, Hongxing Li

**Affiliations:** 1 Department of Neurosurgery, Shengli Oilfield Central Hospital, Dongying, Shandong Province 257000, P. R. China; 2 Clinical Teaching and Research Group, Shandong Shengli Vocational College health care Branch, Dongying, Shandong Province, P. R. China

**Keywords:** Laryngeal neuralgia combined with intermediate nerve neuralgia, Trigeminal neuralgia, Rhizotomy

## Abstract

**Background:**

In neurosurgery, the most common type of facial and pharyngeal pain is trigeminal neuralgia. In contrast, glossopharyngeal neuralgia is relatively rare, and laryngeal neuralgia is the most rarely observed.

**Case presentation:**

A case of laryngeal neuralgia combined with intermediate nerve neuralgia that was admitted to our hospital in May 2012 was reported here. The patient was a 58-year-old middle-aged female, who experienced 2 years of paroxysmal burning and stabbing pain near the thyroid perichodrium, in the skin covering the right front side of the neck, and deep in inner ear.

**Conclusion:**

The surgical treatment plan similar to that for glossopharyngeal neuralgia could be applied if laryngeal neuralgia is associated with glossopharyngeal neuralgia and intermediate neuralgia or if no obvious improvement is achieved with the above mentioned treatment approaches.

## Background

In neurosurgery, the most common type of facial and pharyngeal pain is trigeminal neuralgia. The unbearable pain caused huge trouble to the patient, and seriously affected the quality of patients’ life. The most common type is vagus nerve neuralgia, and glossopharyngeal neuralgia. In contrast, laryngeal, intermediate nerve neuralgia is relatively rare, and laryngeal combined with intermediate nerve neuralgia is the most rarely observed. Because there is no basic pathophysiology conclusion about this neuropathic pain, so many treatment methods existed. The treatment about laryngeal combined with intermediate nerve neuralgia is also very difficult. We reported here a case of laryngeal combined with intermediate nerve neuralgia that was admitted to our hospital in May 2012.

## Case presentation

Written informed consent for participation in the study was obtained from the participant in this study. The patient was a 58-year-old middle-aged female, who experienced 2 years of paroxysmal burning and stabbing pain near the thyroid perichodrium, in the skin covering the right front side of the neck, and deep in inner ear. Touching the skin in front of the neck or swallowing could provoke the pain. It initially only affected areas near the thyroid perichodrium and the skin covering the right front side of the neck, and it was later deteriorated and also affected areas deep in the inner ear. Each episode of pain lasted a few seconds to a few minutes, and in severe cases could last for hours. The pain was severe, and when evaluated using the ‘Wong-Baker Faces pain rating scale’ [1], the patient was determined to have a pain scale of Face 10 hurts worst. The patient had frequent episodes of pain. Oral administration of carbamazepine could achieve partial remission in early stages of the disease, but later had only poor effects. The patient could not engage in any work during the episodes of pain, and even considered suicide. To ease the pain, the patient had to take a daily carbamazepine dosage up to 2.0 g. Nervous system examination did not reveal positive signs. Enhanced scan of Magnetic resonance tomoangiography (MRTA) did not reveal any clear responsible vascular compression in the trigeminal nerve, the glossopharyngeal nerve, and the vagus nerve. The patient previously received regional blockage of the right laryngeal nerve in other hospitals, and the pain in the thyroid perichodrium and the skin covering the right front side of the neck was relieved, whereas the pain deep in the ear did not show apparent relief. Therefore, we reasoned that it might be due to laryngeal neuralgia combined with intermediate nerve neuralgia. After admitted to our department, we utilized a surgical plan that was often used for the treatment of glossopharyngeal neuralgia and performed the rhizotomy procedure sectioning the intermediate nerve, the glossopharyngeal nerve and the upper 1st to 2nd rootlets of the vagus nerve via the keyhole retrosigmoid approach. The disease was cured after the surgery, and the patient was pain-free in the follow-up examinations performed 6 months and 12 months after the surgery.

The surgical methods were as follows: the patient was placed under general anesthesia and was in the left lateral position, with the head lowered 15° and rotated 10° toward the left. The neck of the patient was slightly flexed forward so that the ipsilateral mastoid process was roughly parallel to the plane of the operating table. A suboccipital transverse incision with a length of 3.5 cm was made starting from the root of mastoid process. The occipital bone window had a diameter of 1.5 cm, with the anterior margin adjacent to the sigmoid sinus. A “**⟂**” shaped incision was made to open the dura mater, and all subsequent procedures were carried out under a surgical microscope. The cerebellar hemispheres were gently retracted in order to fully release the cerebrospinal fluid and reveal the posterior cranial nerves. The arachnoid mater at the roots of the cranial nerves was cut open and the cerebellar flocculus was retracted with a brain spatula. Meanwhile, the position of the patient's head and (or) the angle of the microscope optical axis were adjusted, and the facial and acoustic nerves were exposed. The auditory nerve was gently pulled aside with a detacher, and the intermediate nerve between the auditory nerve and the facial nerve was then exposed and severed using the microsurgery scissors. The glossopharyngeal nerve above the posterior cranial nerve was identified and severed. The vagus nerve rootlets were identified, which included eight rootlets arranged from caudal to rostral. The caudal 1st and 2nd vagus nerve rootlets were sectioned. Neither vascular compression of the nerves nor arachnoid thickening and adhesion were observed. After verifying that there was no bleeding, conventional craniotomy closure was performed. Suture removal was performed 7 days after the surgery and the patient was then discharged. The paroxysmal burning and stabbing pain near the thyroid perichodrium, in the skin covering the right front side of the neck, and deep in the right inner ear disappeared after the surgery.

## Discussion

Laryngeal neuralgia is extremely rare, there was no large-scale case analysis of laryngeal neuralgia, and only a few isolated cases have been reported [2]. This disease presents symptoms resembling those of glossopharyngeal neuralgia without any typical symptoms. It can be distinguished from glossopharyngeal neuralgia based on the facts that the pain is originated from the throat, in a small portion of patients the sensation of pain radiates to the ipsilateral jaw and ear, pain is usually caused by sneezing or swallowing, the degree of pain is severe, patients usually are afraid to speak due to pain, and the site triggering the pain is located in the anterior piriform recess. Since the etiology of laryngeal neuralgia is not fully understood and there are no standard treatment methods for this disease, we are not discussing here the diagnosis and treatment principles of this disease. Instead, we hoped to introduce one case of laryngeal neuralgia that we treated successfully with a surgical method similar to that used to treat glossopharyngeal neuralgia and to share our experience with everyone.

Laryngeal nerve is a branch of the vagus nerve arising from the nodose ganglia. It descends to the plane of hyoid bighorn and divides into the internal sensory branch and the external motor branch. Lesions occurring at any point in the laryngeal neural pathways could lead to the possibility of laryngeal neuralgia. The etiology of this disease is not clear, and the reported causes of the disease include deviation of the hyoid bone [3] and trauma [4]. In addition, post-traumatic laryngeal neuralgia occurred in patients undergone tonsil resection, suggesting the possibility that post-surgical scars lead to laryngeal nerve adhesions.

Similar to patients with diseases such as trigeminal neuralgia and glossopharyngeal neuralgia, patients with laryngeal neuralgia can be treated with oral administration of carbamazepine to relieve symptoms, while others have reported cases cured by carbamazepine treatment alone [5,6]. For patients who had poor effects with oral administration of carbamazepine, regional nerve blockage [7] or laryngeal neurectomy can also be utilized to achieve therapeutic effects. Intermediate nerve Neuralgia is also called geniculate neuralgia, main performance are electric shock pain in the distribution area of intermediate nerve, such as intermittent pain in the deep of ear, and Electric shock tingling pain, these pains are exactly as same as tympanic nerve neuralgia which is branch of glossopharyngeal nerve. The pains are all on one side, and completely disappear during intermitten perioud [2]. Stimulating the external auditory canal, swallowing, or speaking can trigger pain. Clark and Taylor [8] first reported one case cured by severing off intermediate nerve under the pillow. Fanzhong [9] reported a case by severing off intermediate nerve from the back of the lost path [1,4,10]. When laryngeal nerve neuralgia combined with intermediate nerve neuralgia occur at the same time, only severing off laryngeal nerve cannot reveal the pain thoroughly, because the glossopharyngeal nerve, intermediate nerve and the upper 1st to 2nd rootlets of the vagus nerve are around, so we need to sever off the glossopharyngeal nerve, intermediate nerve and the upper 1st to 2nd rootlets of the vagus nerve together.

## Conclusions

The patient in our study was first treated with drugs such as carbamazepine at early stages of the disease. But oral medication was ineffective, and the largest dose of carbamazepine was increased to 2.0 g/day. The regional blockage of laryngeal nerve was subsequently performed, which also led to transient treatment effect, followed shortly with disease recurrence. After recurrence, the patient showed severe unbearable pain, and the patient also showed symptoms of intermediate nerve neuralgia. We thus performed rhizotomy targeting the glossopharyngeal nerve and the upper 1st to 2nd rootlets of the vagus nerve via the retrosigmoid approach, which is similar to the treatment plan for glossopharyngeal neuralgia. The symptoms were relieved after the surgery. It has been reported that sectioning of the intermediate nerve can be performed for patients with pain associated with deep inner ear with the cure rate reaching 80% and the efficiency above 90% [11]. We therefore severed the intermediate nerve of the affected side. Laryngeal neuralgia is a rare disease. Once it is diagnosed based on the existence of throat pain and the absence of other disease, it can be treated first with medication, followed by regional blockage of laryngeal nerve or neurotomy. The surgical treatment plan similar to that for glossopharyngeal neuralgia could be applied if laryngeal neuralgia is associated with glossopharyngeal neuralgia and intermediate neuralgia or if no obvious improvement is achieved with the above mentioned treatment approaches.

## Consent

Written informed consent was obtained from the patient for publication of this Case report and any accompanying images. A copy of the written consent is available for review by the Editor of this journal.

## Competing interests

The authors declare that they have no competing interests.

## Authors’ contributions

Study concept and design, and study supervision: KZ and QZ. Acquisition of data, analysis of data and drafting of manuscript: QZ, GH, SY and LW. HL’s contribution to the manuscript is discussion and result analysis. All authors read and approved the final manuscript.

## Pre-publication history

The pre-publication history for this paper can be accessed here:

http://www.biomedcentral.com/1471-2482/14/60/prepub
